# Carotid Artery Geometry Modifications and Clinical Implications after Carotid Artery Stenting

**DOI:** 10.3390/jpm14111091

**Published:** 2024-11-04

**Authors:** Edoardo Pasqui, Bruno Gargiulo, Leonardo Pasquetti, Elisa Lazzeri, Giuseppe Galzerano, Gianmarco de Donato

**Affiliations:** Vascular Surgery Unit, Department of Medicine, Surgery and Neuroscience, University of Siena, 53100 Siena, Italy; bruno.gargiulo@student.unisi.it (B.G.); l.pasquetti@student.unisi.it (L.P.); e.lazzeri3@student.unisi.it (E.L.); giuseppe.galzerano@unisi.it (G.G.); dedonato@unisi.it (G.d.D.)

**Keywords:** carotid artery disease, carotid artery stenting, endovascular treatment, angiography

## Abstract

Background: Carotid artery stenting (CAS) could lead to a modification of the carotid bifurcation geometry with possible clinical implications. This study aimed to clarify the geometrical impact of three carotid stents with different designs on the carotid bifurcation and its clinical consequences. Methods: This was a retrospective single-center study. We included all patients who underwent CAS in a 3-year period. Anatomical changes of the carotid bifurcation were evaluated by reviewing angiographic images. The population was divided into three groups based on the stent implanted: Group 1 (Carotid Wallstent), Group 2 (Roadsaver), and Group 3 (C-Guard). Results: A total of 226 patients were included. The mean age was 77.0 ± 7.4 years and 72.5% (164/226) were male. Three different stents were implanted into three groups: Group 1 (*n* = 131/226, 58%), Group 2 (*n* = 57/226, 25.2%), and Group 3 (*n* = 38/226, 16.8%). The mean pre-stent implantation CCA-ICA angle of the entire population was 155 ± 14.9°, and the post-CAS angle was 167.7 ± 8.7° (*p* = 0.0001). In every subgroup, the difference was statistically different, with the biggest difference registered in Group 2 (−16.1 ± 13.2°). Regarding stent oversizing, there was a significant relationship between CCA oversizing and CCA-ICA angle modification (*p* = 0.006). During follow-up, a total of 14 (6.2%) restenoses were registered. The mean CCA-ICA angle modification in the restenosis group was −9.5 ± 14.4° vs. −12.8 ± 11.9° in the no-restenosis group with no significant statistical differences were outlined (*p* = 0.3). Conclusions: Compared to the Carotid Wallstent and C-Guard, the Roadsaver stent appears to have a lower adaptability to the carotid vascular territory, resulting in a higher CCA-ICA angle modification after implantation, with no impact on the stent restenosis rate.

## 1. Introduction

Carotid artery stenting (CAS) represents an alternative to carotid endarterectomy (CEA) in asymptomatic patients with good life expectancy, favorable anatomy, and plaque morphology [1].

Over time, CAS technology has evolved towards low-profile, atraumatic, and flexible devices. The technological advancement aimed to offer the best compromise of carotid plaque scaffolding and flexibility to respect the original patient’s anatomy. Different types of devices have been proposed, from open-cell to closed-cell devices to the more recent double-layer stent with higher scaffolding properties. This evolution widened the indication for CAS, which can also treat soft and lipid-rich carotid plaques.

The interaction between implantable devices and the receiving arteries could have technical and clinical impacts, especially in terminal vascular territories such as the internal carotid artery (ICA).

The post-procedural modification of the tridimensional carotid artery bifurcation could cause increased or decreased shear stress at the level of the specific stent location, as well as in the coronary district, leading to blood flow turbulence and a higher risk of restenosis [2]. In light of this, devices with higher conformability and flexibility would reduce anatomic alterations and their possible clinical impact. The objective evaluation of the stent adaptability could be challenging but its quantification could be important in terms of stenting performance.

On this particular topic, very few reports have been published at this time, using different methods of investigation (in vivo and in vitro analyses) with variable outcomes.

Thus, this study aimed to evaluate the interaction between different types of carotid stents and the carotid anatomy, focusing on the modification of the angulation between the common carotid artery (CCA) and ICA and its influence on restenosis and ipsilateral cerebral ischemic events.

## 2. Materials and Methods

### 2.1. Population Cohort

All patients who underwent primary CAS from January 2019 to January 2022 were initially included in the analysis. The patients’ demographics, intraoperative data, and postoperative outcomes were collected through hospital charts. All patients were evaluated in the preoperative time with duplex vascular ultrasound in order to assess plaque morphology, plaque extension, and carotid artery diameters. Computed Tomography Angiography (CTA) was performed preoperatively to evaluate the aortic arch configuration, intracerebral vasculature, and carotid plaque characteristics. Indications for revascularization followed the NASCET [3] criteria and duplex ultrasound velocity alterations proposed by the European Society For Vascular Surgery guidelines [1,4]. Dual antiplatelet therapy was assured during the preoperative period and continued for at least 1 month after the index procedure. CAS procedures were performed using a transfemoral approach under local anesthesia via an ultrasound-guided puncture. For all the patients, the position on the surgical table was supine with the head rotated at an angle of 45°to open and maximize the carotid bifurcation view. Moreover, in order to reduce movements, the patient’s head was immobilized. In the case of particular anatomic conditions, the standardized position was modified. Cerebral hemispheric perfusion was continuously assessed using a Near-Infrared Spectroscopy (NIRS) system when possible. Systemic heparinization was reached with intravenous heparin (100 UI/kg) after positioning the 8 Fr introducer sheath in the common femoral artery. All procedures were performed using cerebral protection devices and various stent models. The choice between different types of stents was based on anatomical considerations and device availability. In particular, the composition of the plaque to be treated was the main variable that guided the stent choice. In order to ensure a higher scaffolding property, a double mesh stent was preferred in the case of lipid-rich plaques. Stent dimensions were chosen according to preoperative routine DUS examination and CTA. Percutaneous arterial access hemostasis was achieved via manual compression or closure devices. The total population was divided into three different groups based on the type of stent implanted: Group 1, characterized by the use of the closed-cell stent Carotid Wallstent (Boston Scientific, Santa Clara, CA, USA); Group 2 patients were treated with the double-layer mesh stent Roadsaver (Terumo Corporation, Tokyo, Japan); and Group 3 patients were treated with the double-mesh C-Guard stent (InspireMD Inc., Boston, MD, USA).

### 2.2. Anatomical Consideration

**Figure 1:**
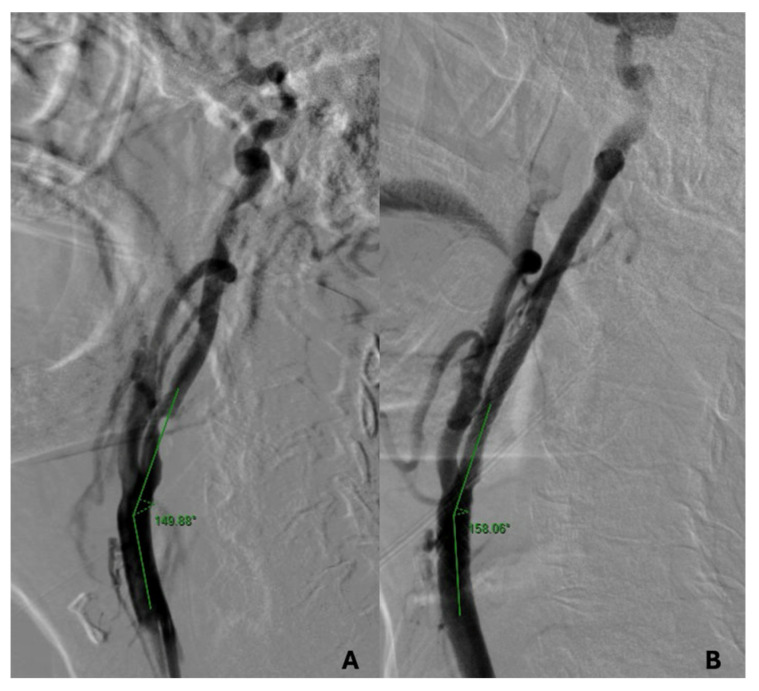
Example of carotid bifurcation analysis during CAS procedure with the implantation of Roadsaver stent. (**A**) Pre-stenting CCA-ICA angle; (**B**) post-stenting CCA-ICA angle.

The carotid axis geometry was obtained by calculating the angle between the central axis of the common carotid artery and the central axis of the proximal segment of the ICA in the predefined projection. The calculation was performed in all patients before and after stent release in order to evaluate possible geometrical differences (Figure 1A,B). Post-stent implantation images were obtained after the removal of any guidewires or embolic protection devices in order to reduce any confounders. Angulation changes were also evaluated based on the type of carotid plaque (hypo-, iso-, and hyperechoic plaques) and the rate of stent oversizing with respect to the common carotid artery (CCA) and ICA.

### 2.3. Outcomes

The primary endpoint was defined as the absolute change in the CCA-ICA angle after stent deployment for the three different stents implanted. Secondary endpoints were anatomical angulation changes based on plaque constitution and carotid stent oversizing. Moreover, the risk of any neurological event or death within the first 30 days after the intervention was also considered as a secondary endpoint. Secondary late outcomes included the rate of restenosis during follow-up with particular attention to the eventual relationship with CCA-ICA changes after stent deployment. Neurological complications were classified as follows: TIA, minor stroke, and major stroke. Restenosis was defined as a narrowing of the treated carotid artery ≥50%, highlighted by DUS or CTA, considering the revised velocity criteria for carotid stenting [5]. The ethical committee of the hospital was informed of the non-experimental design of the retrospective investigation and endorsed the study.

### 2.4. Statistical Analysis

Categorical data were reported as numbers and percentages. Means (±standard deviation) and medians were used to analyze continuous variables. Student’s two-tailed *t*-test and ANOVA were used when applicable for within- and between-group comparisons for continuous data, and categorical variables were compared using Fisher’s exact test. Linear regression analysis was used to evaluate the relationship between stent oversizing and CCA-ICA angulation modification. In evaluating concordance between imaging evaluations, inter- and intra-observer variabilities were assessed using the Cohen’s kappa test of concordance. A k value of 0.61–0.80 and 0.81–1.0 indicated good agreement and excellent agreement, respectively. A *p*-value of <0.05 was considered statistically significant. All statistical analyses were performed with GraphPad Prism 8.0 (GraphPad Software Inc., San Diego, CA, USA) and StatPlus Build 7.1.1 (AnalysisSoft Inc., Walnut, CA, USA).

## 3. Results

### 3.1. Baseline Population Characteristics

A total of 226 patients were included in the analysis. The mean age was 77.0 ± 7.4 years and 72.5% (164/226) were male. The full baseline characteristics are listed in Table 1. Most of the patients were asymptomatic; only 6.6% were admitted after an ischemic neurological event (15/226).

The mean carotid artery stenosis was 82.4 ± 8.7. In 56.6% of cases (128/226), the carotid plaque was mostly hyperechoic and mostly located at the level of the bulb/origin of the ICA (57.1%, 129/226)

Three different stents were implanted: the Carotid Wallstent (Group 1, *n* = 131/226, 58%), Roadsaver (Group 2, *n* = 57/226, 25.2%), and C-Guard (Group 3, *n* = 38/226, 16.8%). The patients’ baseline characteristics were similar between the three groups without significant statistical differences (Table 2).

### 3.2. Anatomical Consideration

**Figure 2:**
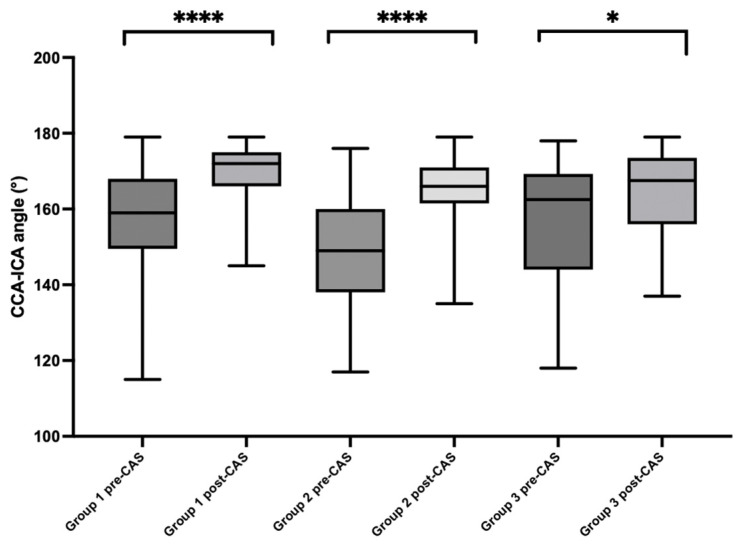
CCA-ICA angle modification after stent implantation among the three subgroups. **** for *p* < 0.001 and * for *p* < 0.05.

Before starting the population analysis, two operators (BG, PE) were trained with 10 CAS imaging evaluations of previous patients that were not included in this study to examine the pre- and post-implantation CCA-ICA angles. The inter-observer variability was very good for the measurement of the CCA-ICA angle: for the overall evaluation, k = 0.91; for the pre-CAS angle, k = 0.92; and for the post-CAS angle, k = 0.89. The intra-observer agreement, evaluated by asking each observer to assess the images twice with a 7-day interval, was excellent (k = 0.94). The anatomical modification of the CCA-ICA angle pre- and post-stent implantation was evaluated. Moreover, a subset of 30 cases were randomly selected and a comparison between the pre-CAS CCA-ICA angle in the preoperative CTA and the pre-CAS CCA-ICA angle in digital subtraction angiography was made. The results were, respectively, 154.6 ± 13.2° vs. 154.1 ± 13.1° (*p* = 0.8), confirming that this method of imaging analysis is reliable. The mean pre-stent implantation CCA-ICA angle of the entire population was 155 ± 14.9°, and the post-CAS angle was 167.7 ± 8.7° (*p* = 0.0001). Analysis stratification by the type of stent implanted was performed. The pre- and post-CAS angles were, respectively, 156.8 ± 13.8° and 169.6 ± 7.1° (*p* = 0.0001) in Group 1, 149.7 ± 14.8° and 165.8 ± 8.4° in Group 2 (*p* = 0.0001), and 156.5 ± 16.9° and 164.2 ± 11.9° in Group 3 (*p* = 0.02). See Figure 2 for a graphical depiction of the data.

**Figure 3:**
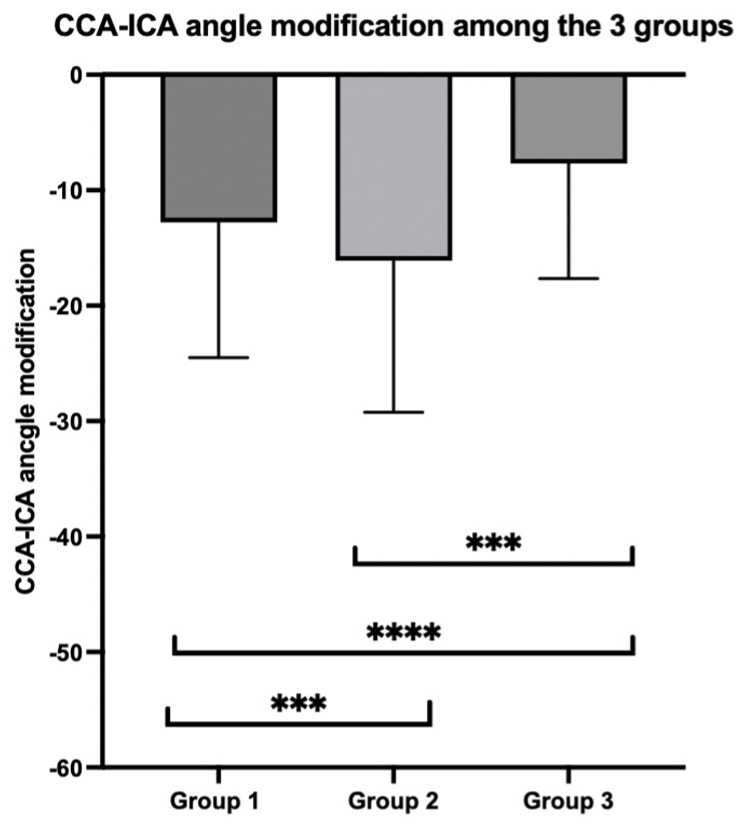
Mean differences in CCA-ICA angle modification among the three subgroups. **** for *p* < 0.001 and *** for *p* < 0.01.

In every subgroup, the difference was statistically different, with the biggest difference in Group 2 (−16.1 ± 13.2°) and the smallest one was found in Group 3 (−7.7 ± 9.9°) (Figure 3).

**Figure 4:**
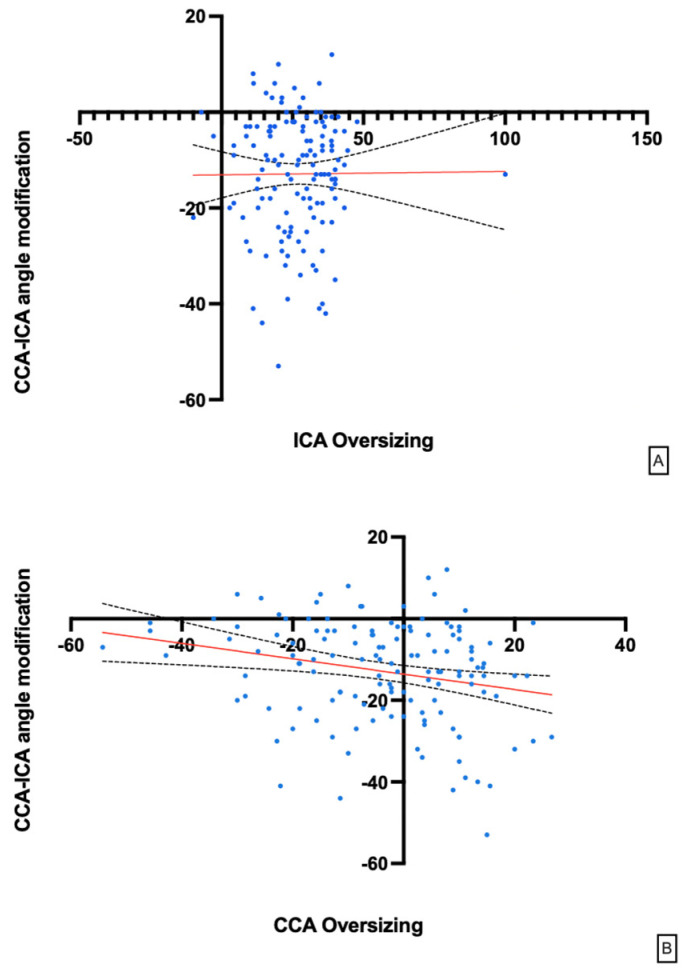
(**A**) Linear regression analysis of CCA-ICA angle modification and ICA stent oversizing; (**B**) linear regression analysis of CCA-ICA angle modification and CCA stent oversizing.

The mean differences between the three groups were significantly different (*p* = 0.03) (Table 2). We also evaluated the potential influencing impact of the type of carotid plaque in terms of the stent accommodation and consequent CCA-ICA angle modification. As listed in Table 1, the majority of plaques were found to be calcified with a hyperechoic aspect during the DUS examination (128/226, 56.6%). The pre- and post-stent implantation CCA-ICA angles in hyperechoic plaques (123/226) were, respectively, 154.6 ± 14.7° and 167.7 ± 9.1° (*p* = 0.001), while in non-hyperechoic plaques, they were 154.5 ± 15.8° and 167.1 ± 9.4° (*p* = 0.001). The statistical analysis did not find a significant difference in terms of CCA-ICA angle modification for the different plaque compositions (*p* = 0.9). The investigation was also focused on the role of stent oversizing for the CCA and ICA diameters. The mean CCA and ICA diameters in the entire population were, respectively, 8.4 ± 0.9 mm and 6.0 ± 0.7. Among the three subgroups, there were no differences (see Table 3 for full details). The mean CCA and ICA oversizing rates were 8.9 ± 36.8% and 26.6 ± 13.2%, respectively. Using the linear regression method, the oversizing rates were related to the CCA-ICA angle modification after stent implantation. The analysis highlighted that there was no significant relationship between ICA oversizing and angle modification (slope equation Y = 0.007007 * X − 13.08, *p* = 0.9). On the other hand, CCA oversizing had a mild impact on angle modification, which was statistically significant (slope equation Y = −0.1886 * X − 13.60, *p* = 0.006) (Figure 4A,B).

### 3.3. Clinical Implications

During the 1-month follow-up, five ipsilateral ischemic events were registered (5/226, 2.2%). All of them were TIAs with complete recovery. Two deaths were recorded: one due to a cerebral contrast medium reaction and severe cerebral edema; another due to post-procedural cerebral hemorrhage in a patient taking a direct oral anticoagulant. At a mean follow-up of 27.6 ± 13.5 months, 27 (11.9%) deaths were registered, but none of them were related to neurological events. A total of 14 (6.2%) restenoses were registered during clinical follow-up; of them, 7 belonged to the Carotid Wallstent group (7/131, 5.359), 6 to the Roadsaver group (6/57, 10.5%), and the residual one to the C-Guard group (1/38, 2.6%). The mean CCA-ICA angle modification in the restenosis group was −9.5 ± 14.4° vs. −12.8 ± 11.9° in the no-restenosis group, with no statistically significant differences outlined (*p* = 0.3).

## 4. Discussion

The study investigated the anatomical changes to the CCA-ICA segment after a CAS procedure.

Among the three different stent types, Carotid Wallstent, Roadsaver, and C-Guard, Roadsaver had the most significant impact on the carotid anatomy, resulting in the most important alterations to the CCA-ICA angle. Moreover, in our experience, stent oversizing with respect to the CCA diameter seemed to influence the carotid anatomy straightening. The CCA-ICA angle post-CAS modification was not related to the occurrence of CAS restenosis during follow-up.

In the last three decades, different stents have been proposed to improve CAS performance and to widen the CAS clinical indications. From the very flexible and adaptable open-cell nitinol stents towards closed-cell stents and finally, new generation double-layer devices, endovascular carotid procedures have evolved. These modifications have been developed to increase the scaffolding properties of the devices, reducing the risk of micro- and macro-embolization, and allowing for the treatment of soft and lipid-rich plaques [6,7,8,9,10,11].

The conformability of implantable carotid devices has been investigated since their exploratory use. Tanaka et al. in 2004 reported an efficiency analysis based on an artificial carotid model [12].

They compared different stents: stents braided from continuous filaments and segmented nitinol stents with an open-cell design. The results were significant: the implantation of braided stents with continuous filaments induced considerable straightening effects on the bifurcation angle, as well as on the curves of the internal carotid artery. Segmented designs of modular nitinol stents complied better with vascular tortuosity with increased adaption between the stent and the model. More recently, an assessment and comparison with manufacturer data was performed for different carotid stent types [13]. In terms of flexibility, closed-cell stents had a lower flexibility in comparison with open-cell stents. Roadsaver, due to its typical stent design, has low flexibility compared to the C-Guard stent, which is rigid when mounted on the delivery system and very flexible in its expanded state. Recently, CCA-ICA angle modifications were evaluated for Wallstent, Roadsaver, and C-Guard. The analysis reported that no statistical difference in the axial vessel geometry between the basal and postprocedural angles was found for C-Guard; on the other hand, Wallstent and Roadsaver showed a significant modification between the pre- and post-angles [14]. Our experience is in line with the previous report: C-Guard was the most conformable stent among the ones included in the analysis, with a lower impact on the carotid anatomy. We also studied the clinical side in the analysis, evaluating the relationship between carotid straightening and the risk of a follow-up restenosis. No significant differences emerged regarding the modification of the CCA-ICA angle between the restenosis and no-restenosis group; in other words, this kind of modification did not seem to be associated with a lower or higher risk of restenosis. Fluid and hemodynamic analyses could clarify the impact of carotid anatomy modification on the risk of increased restenosis and neo-intimal hyperplasia. Very few experiences are reported in the literature regarding CAS hemodynamics. Recently, Ren S et al. reported an exploratory study focused on the hemodynamic impact of carotid endarterectomy end stenting in patients with a diagnosis of carotid web, highlighting that CAS has more impact on the blood flow at the level of carotid siphon due to an increase in kinetic energy [15]. Moreover, computational studies found that closed-cell stents may pose a higher risk for in-stent restenosis than the open-cell stent design due to slightly larger areas of low wall shear stress, an elevated oscillatory index, and high relative residence time [16].

We also investigated the role of stent oversizing on CCA-ICA angulation modification. The literature has not investigated this particular field even if stent oversizing has been related to the risk of restenosis. We highlighted that a bigger oversizing of the stent with respect to the CCA has an impact on CCA-ICA angle straightening; on the other hand, ICA oversizing does not seem to result in the same modification. There is no literature data regarding these particular findings, but in the past, Piamsomboon et al. reported the first evaluation of whether stenting oversizing is related to late luminal loss. The outcomes did not highlight a direct correlation between stenting oversizing and late luminal loss and follow-up restenosis [17]. This was also confirmed in a canine model in which 30–40% stent oversizing did not result in excessive neointimal hyperplasia with respect to normal-sized stents [18].

In addition to the very few reports already published, this work is a first step in understanding the impact of carotid artery stents on a diseased carotid district. The future perspectives should focus on defining a personalized treatment protocol for different types of anatomy and carotid artery disease. Of course, the next step would be enriched by a prospective fluidodynamic investigation in order to consider the role of carotid artery anatomy modifications and blood-flow alterations in clinical complications such as hemodynamic restenosis and/or ipsilateral post-CAS cerebral ischemic events.

### Limitations

The main limitation is the retrospective nature of the study and the low number of patients in the cohort.

In addition, the angiographic angle estimation could have had measurement discrepancies due to the lack of a tridimensional view. CT-Angio is not routinary performed after CAS procedures due to the radiation exposure, contrast medium need, and the wide acceptance of DUS for carotid disease follow-up. Lastly, as a retrospective study, we did not have data on the very early hemodynamic changes after the CAS that could help in understanding the real clinical impact of the carotid geometry changes.

## 5. Conclusions

The C-Guard stent represents the most conformable device for CAS with the best interplay with carotid artery anatomy compared to the Roadsaver and Carotid Wallstent. Moreover, carotid plaque morphology and ICA stent oversizing seem to not influence stent adaptability while CCA stent oversizing correlates with CCA-ICA angle straightening. Lastly, carotid artery straightening does not imply a higher risk of restenosis.

## Figures and Tables

**Table 1:** Baseline characteristics of the entire population and their distribution among the three groups.

Variable	Whole Population (*n* = 226)	Group 1 (*n* = 131)	Group 2 (*n* = 57)	Group 3 (*n* = 38)	*p* Value
Age (mean ± SD)	77 ± 7.4	76.8 ± 7.4	77.3 ± 7.4	76.9 ±7.5	0.7
Male (*n*, %)	164 (72.5%)	89 (68.0%)	45 (78.9%)	30 (78.9%)	0.2
Hypertension (*n*, %)	192 (84.9%)	111 (84.7%)	48 (84.2%)	33 (86.8%)	0.9
Dyslipidemia (*n*, %)	175 (77.4%)	105 (80.2%)	39 (68.4%)	32 (84.2%)	0.1
CAD (*n*, %)	58 (25.6%)	37 (28.2%)	11 (19.3%)	10 (26.3%)	0.6
PAD (*n*, %)	16 (2.6%)	8 (6.1%)	6 (10.5%)	2 (5.3%)	0.6
CKD (*n*, %)	22 (9.7%)	11 (8.4%)	5 (8.8%)	6 (15.8%)	0.4
AF (*n*, %)	24 (10.6%)	14 (10.7%)	3 (5.2%)	7 (18.4%)	0.1
DM (*n*, %)	72 (31.8%)	42 (32.1%)	18 (31.6%)	12 (31.6%)	0.9
Symptomatic (*n*, %)	15 (6.6%)	3 (2.3%)	3 (5.3%)	9 (23.7%)	0.0001
Stenosis (mean ± SD)	82.4 ± 8.7	81.6 ± 9.1	83.4 ± 9.4	83.3 ± 7.1	0.8
Hyperechoic plaque (*n*, %)	128 (56.7%)	83 (63.4%)	30 (52.6%)	15 (39.5%)	0.09
Hypoechoic plaque (*n*, %)	51 (22.6%)	23 (17.6%)	15 (26.3%)	13 (34.2%)	0.09
Isoechoic plaque (*n*, %)	47 (20.7%)	25 (19.1%)	12 (21.1%)	10 (26.3%)	0.09
Plaque localization (*n*, %) Bulb ICA Bulb origin I° part of ICA	14 (6.2%) 129 (57.1%) 87 (38.5%)	12 (9.1%) 73 (55.7%) 50 (38.2%)	1 (1.8%) 32 (56.1%) 25 (43.9%)	1 (2.6%) 24 (63.2%) 12 (31.2%)	0.1 0.2 0.2
Irregular profile (*n*, %)	83 (36.7%)	44 (33.6%)	20 (35.1%)	19 (50%)	0.2
Ulceration (*n*, %)	26 (11.5%)	12 (9.1%)	7 (12.3%)	7 (18.4%)	0.3
Arch type (*n*, %) Type I Type II Type III Bovine	141 (62.4%) 45 (19.9%) 40 (17.7%) 13 (5.8%)	90 (68.7%) 22 (16.8%) 19 (14.5%) 9 (6.9%)	33 (57.9%) 13 (22.8%) 11 (19.3%) 2 (3.5%)	18 (47.3%) 10 (26.3%) 10 (26.3%) 2 (5.3%)	0.1 0.1 0.1 0.2

Abbreviations: CAD, Coronary Artery Disease; PAD, Peripheral Artery Disease; CKD, Chronic Kidney Disease; AF, Atrial Fibrillation; DM, Diabetes Mellitus; ICA, internal carotid artery.

**Table 2:** CCA-ICA angulation before and after stent implantation for the whole study population and the three subgroups.

Variable	Whole Population (*n* = 226)	Group 1 (*n* = 131)	Group 2 (*n* = 57)	Group 3 (*n* = 38)	*p* Value
Pre-stenting CCA-ICA angle	155 ± 14.9	156.8 ± 13.8	149.7 ± 14.8	156.5 ±16.9	0.6
Post-stenting CCA-ICA angle	167.7 ± 8.7	169.6 ± 7.1	165.8 ± 8.4	164.2 ± 11.9	0.5
*p* value	0.0001	0.0001	0.0001	0.02	

Abbreviations: CCA, common carotid artery; ICA, internal carotid artery.

**Table 3:** Anatomical carotid bifurcation variable and stent device characteristics.

Variable	Whole Population (*n* = 226)	Group 1 (*n* = 131)	Group 2 (*n* = 57)	Group 3 (*n* = 38)	*p* Value
CCA diameter	8.4 ± 0.9	8.4 ± 0.9	8.6 ± 1.1	8.2 ± 0.6	0.2
ICA diameter	6.0 ± 0.7	6.0 ± 0.8	6.1 ± 0.8	5.8 ± 0.7	0.3
Stent diameter	8.2 ± 0.8	8.2 ± 1.0	8.2 ± 0.4	8.1 ± 0.8	0.4
Stent length	33.1 ± 6.2	35.0 ± 5.5	26.6 ± 3.7	35.7 ± 5.1	0.06

Abbreviations: CCA, common carotid artery; ICA, internal carotid artery.

## Data Availability

The raw data supporting the conclusions of this article will be made available by the authors on request due to privacy restrictions.
